# Zinc regulation of chlorophyll fluorescence and carbohydrate metabolism in saline-sodic stressed rice seedlings

**DOI:** 10.1186/s12870-024-05170-w

**Published:** 2024-05-27

**Authors:** Kun Dang, Jinmeng Mu, Hao Tian, Dapeng Gao, Hongxiang Zhou, Liying Guo, Xiwen Shao, Yanqiu Geng, Qiang Zhang

**Affiliations:** 1 Agronomy College, Jilin Agricultural University, Changchun, 130118 China; 2 Key Laboratory of Germplasm Innovation and Physiological Ecology of Coldland Grain Crops, Ministry of Education, Harbin, 150000 China

**Keywords:** Rice, Saline-sodic stress, Zinc, Chlorophyll fluorescence, Carbohydrate metabolism

## Abstract

Saline-sodic stress can limit the absorption of available zinc in rice, subsequently impacting the normal photosynthesis and carbohydrate metabolism of rice plants. To investigate the impact of exogenous zinc application on photosynthesis and carbohydrate metabolism in rice grown in saline-sodic soil, this study simulated saline-sodic stress conditions using two rice varieties, 'Changbai 9' and 'Tonghe 899', as experimental materials. Rice seedlings at 4 weeks of age underwent various treatments including control (CT), 2 μmol·L^−1^ zinc treatment alone (Z), 50 mmol·L^−1^ saline-sodic treatment (S), and 50 mmol·L^−1^ saline-sodic treatment with 2 μmol·L^−1^ zinc (Z + S). We utilized *JIP*-test to analyze the variations in excitation fluorescence and MR820 signal in rice leaves resulting from zinc supplementation under saline-sodic stress, and examined the impact of zinc supplementation on carbohydrate metabolism in both rice leaves and roots under saline-sodic stress. Research shows that zinc increased the chloroplast pigment content, specific energy flow, quantum yield, and performance of active PSII reaction centers (*PI*_ABS_), as well as the oxidation (*V*_OX_) and reduction rate (*V*_red_) of *PSI* in rice leaves under saline-sodic stress. Additionally, it decreased the relative variable fluorescence (*W*_K_ and *V*_J_) and quantum energy dissipation yield (*φ*_DO_) of the rice. Meanwhile, zinc application can reduce the content of soluble sugars and starch in rice leaves and increasing the starch content in the roots. Therefore, the addition of zinc promotes electron and energy transfer in the rice photosystem under saline-sodic stress. It enhances rice carbohydrate metabolism, improving the rice plants’ ability to withstand saline-sodic stress and ultimately promoting rice growth and development.

## Introduction

Soil salinization is a significant abiotic stress that affects global agriculture and impacts plant growth and development [1]. In China, the extent of saline-alkali soil covers approximately 99.13 million hm^2^. The Songnen Plain(42°30–51°20 N and 121°40–128°30 E) alone has an area of 3.73 million hm^2^ of saline-sodic land, making it one of the world's three major areas with this type of soil [2]. The soil in this region contains a significant amount of soluble salt, with NaHCO_3_ and Na_2_CO_3_ being the main salt components. The pH values of the soil in this area are mostly higher than 8.5. The Songnen Plain is one of China's major grain-producing regions; however, its crop yields are severely limited due to the presence of saline-sodic soils. The high concentration of salt ions in saline-sodic soil not only reduces the osmotic potential of the soil, leading to osmotic stress in crops, but also disrupts the delicate ion balance within crop cells as salt ions like Na^+^ and Cl^−^ enter, resulting in ion stress [3]. Additionally, the high pH levels facilitate salt ion absorption by crops, but hinder the absorption of essential nutrient ions such as zinc, iron, calcium, and magnesium, thereby impeding their growth and development [4]. Consequently, alleviating saline-sodic stress, improving crop nutrient absorption, and promoting crop growth and development are significant challenges in agriculture within this region.

Zinc, a micronutrient crucial for crop growth and development, acts as a cofactor for numerous enzymes involved in key physiological and biochemical processes like photosynthesis and hormone synthesis [5]. Zinc in the soil is primarily absorbed by the plant in its Zn^2+^ state. The root cells absorb Zn^2+^ through both passive and active transport via the cell membrane [6]. The uptake and transport of zinc in plants can be influenced by external factors, including saline-sodic conditions. Research indicates a strong correlation between soil saline-sodic concentration, and zinc availability [7]. It has been observed that as the pH levels rise, the amount of zinc accessible to wheat plants decreases notably, particularly in soils rich inwith high bicarbonate content [8]. However, zinc deficiency has significant implications for crop growth and photosynthesis. Research indicates that insufficient zinc levels can result in a decrease in chloroplast count, structural damage to chloroplasts, reduced chlorophyll levels, and diminished photosynthetic activity [9]. Inadequate zinc supply can also lower the maximum quantum efficiency of PSII, disrupt plant chloroplast ultrastructure, decrease carbonic anhydrase activity, and impair photosynthesis [10, 11]. Moreover, zinc deficiency hampers corn leaf photosynthesis, reduces soluble sugar content in corn leaves, and ultimately impacts yield formation [12]. Rice is one of the main food crops in the Songnen Plain, Zinc deficiency, resulting from saline-sodic soil, can impede rice growth and photosynthesis, leading to reduced rice yield. Hence, it is vital to investigate the potential of zinc supplementation through foreign aid in increasing zinc levels in saline-sodic soil, alleviating saline-sodic stress, and ultimately enhancing rice growth and photosynthesis.

Photosynthesis is the fundamental process behind plant growth and development, as it supplies the necessary materials and energy for their progress [13]. Chlorophyll is a key light-absorbing molecule that provides valuable information about the structure, conformation, and function of the photosynthetic apparatus through its fluorescence [14]. In recent years, rapid chlorophyll a fluorescence assays have gained popularity due to their non-destructive, sensitive, fast, and easy operation [15]. These assays provide detailed information on the status and function of *PSII* reaction centers, light-trapping antenna complexes, and *PSII* donor/acceptor side [16]. They have been widely used to analyze crop heat stress [17], saline-sodic stress [18, 19], and other abiotic stress responses [20]. Research has shown that abiotic stress can damage the photosynthetic structure of crop leaves, leading to decreased photophase activity and electron transfer efficiency [21]. This disruption in the balance of reactive oxygen species (ROS) can have a negative impact on crop growth [22]. It has also been found that environmental stress can significantly degrade the D_1_ protein in plant leaves, causing damage to both the donor and recipient sides of *PSII* [23]. Furthermore, stress conditions can hinder the uptake of CO_2_ by crops, while a limited supply of CO_2_ can obstruct the transport, distribution, and utilization of photosynthates. Research indicates that under saline-sodic stress, the combination of ionic toxicity and osmotic stress can cause stomatal closure in rice leaves, thereby affecting the absorption of carbon dioxide [24]. Studies also have demonstrated that saline-sodic stress can impede the uptake of carbon dioxide by influencing crop *PSII*, cytochrome (Cyt *b*_*6*_*/f*) complexes, and electron transfer processes, consequently affecting the production of photosynthetic products [25]. Saline-sodic stress limits light energy in plants, hindering assimilate production and leading to an accumulation of soluble sugars and starch in the source leaves. This accumulation results in feedback inhibition of photosynthesis [26]. Furthermore, saline-sodic stress decreases the carbon content in carbon sink tissues, leading to a reduction in energy supply that impacts crop growth and development [27]. Therefore, enhancing chlorophyll fluorescence parameters in rice under saline-sodic stress and balancing assimilate source-sink conversion are effective strategies for promoting rice growth.

Multiple recent studies have highlighted the significant role of zinc in enhancing plant growth and photosynthesis [28, 29]. However, there is a scarcity of research on the impact of zinc on photosynthetic fluorescence and carbohydrate metabolism in saline-sodic rice. Therefore, this study utilized a NaCl: Na_2_SO_4_: Na_2_CO_3_: NaHCO_3_ of 1:9:1:9 to mimic saline-sodic stress conditions [30]. By employing the *JIP* test in conjunction with chlorophyll fluorescence and the 820 nm technique, the study investigated the effects of zinc supplementation on photosynthetic electron transport chain and non-structural carbohydrate metabolism in rice leaves and roots. Our hypothesis: The addition of exogenous zinc could improve photosynthetic fluorescence parameters in rice under saline-sodic conditions, regulate assimilate source conversion, and ultimately bolster the growth and development of rice.

## Material and methods

### Experimental design

This study utilized two rice (*O.sativa-ssp.japonica*) cultivars: 'Changbai 9' (Changbai No. 9 was developed through a breeding program at the Rice Research Institute of Jilin Academy of Agricultural Sciences in Jilin Province. It was created using Jijing 60 as the female parent and Northeast 125 as the male parent. Initially known as Ji 89–45) and 'Tonghe 899' (Tonghe 899 was developed through a breeding program at Tonghua Academy of Agricultural Sciences, using Y348 as the maternal parent and Ji 01–125/Tonghe 830 as the paternal parent. Initially known as Tonghe 10–8019), with the latter being more sensitive to saline-sodic stress. The test materials consisted of uniformly disinfected rice seeds, treated with a 5% sodium hypochlorite solution for 10 min. The seeds were rinsed with deionized water, germinated in the dark at 30 °C for 48 h, followed by growth in light at the same temperature for another 48 h. Subsequently, the germinated seedlings were moved to a 1/2 nutrient solution for 3 days, before being transferred to a full nutrient solution for a period of 3 weeks. For the upcoming treatments, select rice seedlings that exhibit consistent growth. These treatments include the control group (CT), the zinc alone treatment (Z), the saline-sodic treatment (S), and the saline-sodic zinc treatment (Z + S). The seedlings were subjected to saline-sodic and zinc treatment for 7 days before being sampled for determination. The hydroponic experiment was conducted in a controlled environment room with artificial climate conditions. The photoperiod was set at 14 h of light (28 °C) and 10 h of darkness (22 °C). Additionally, the relative humidity was maintained at approximately 70%. The nutrient solution used in this study was prepared based on the Hoagland nutrient solution preparation method. The composition Hoagland full-concentration nutrient solution is detailed in Table 1. To ensure optimal nutrient availability, the solution was refreshed every three days. In this study, a simulated saline-sodic stress was created using a NaCl: Na_2_SO_4:_ Na_2_CO_3_: NaHCO_3_ ratio of 1:9:1:9, with a saline-sodic concentration of 50 mmol L^−1^, pH of 8.5, and conductivity of 3673 µs cm^−1^ [30]. ZnSO_4_·7H_2_O serves as the primary source of zinc, with an optimal concentration of 2 μmol L^−1^. This concentration was determined through previous screening experiments. To maintain a pH of 5.5 in the nutrient solution, we adjusted with 0.2 mol L^−1^ H_2_SO_4_ or 1 mol L^−1^ KOH every two days.

**Table 1:** Nutrient solution elements and concentration

Element	Salt	Concentration (mg L^−1^)	Element	Salt	Concentration (mg L^−1^)
N	NH_4_NO_3_	91.4	Mn	MnCl_2_·4H_2_O	1.5
P	NaH_2_PO_4_·4H_2_O	40.3	Mo	(NH_4_)_6_Mo_7_O_2_·4H_2_O	0.074
K	K_2_SO_4_	71.4	B	H_3_BO_3_	0.934
Ca	CaCl_2_	88.6	Si	Na_2_SiO_3_·9H_2_O	56.8
Mg	MgSO_4_·7H_2_O	324.0	Fe	FeSO_4_·7H2O	5.57
Cu	CuSO_4_·5H_2_O	0.031	-	-	-

### Sampling and determination

#### Dry weight and leaf water content

The determination of leaf relative water content (RWC) was conducted according to Machado and Paulsen [31]. After subjecting rice seedlings to salt sodium and zinc treatments for 7 days, fully unfolded leaves were chosen. The leaves were briefly rinsed with deionized water and weighed (FW). Subsequently, the leaves were immersed in a container of clean water and sealed for 24 h. After removing the leaves from the water, the surface moisture was wiped off and they were weighed again (SW). Finally, the blades were dried at 80 °C until a constant weight was achieved, and the weight was recorded as DW. This entire process was repeated five times for each treatment. The relative water content (RWC) was calculated using the following formula:

1 $$RWC= (FW-DW) / (SW-DW) \times 100\%$$

#### Determination of chlorophyll content

The method for determining pigment is based on the Gao study [32]. To extract the pigment, a fully unfolded rice leaf is taken, weighing 0.5 g, and placed into a mixture of 15 ml acetone and ethanol (v:v = 1:1). The mixture containing rice leaves is then kept in the dark at 25℃ for 24 h. Subsequently, due to the absorption peaks of chlorophyll a, chlorophyll b and carotenoids at 645 nm and 663 nm and 470 nm. The absorbance values of chlorophyll a, chlorophyll b, and carotenoids at 645 nm and 663 nm and 470 nm were determined using an ultraviolet spectrophotometer (UV-2600, Shimadsu, Japan). The content of chlorophyll a, chlorophyll b, and carotenoids was calculated using the following formula: Subsequently, the absorbance values at 470 nm, 645 nm, and 663 nm are measured using an ultraviolet spectrophotometer (UV-2600, Shimadzu, Japan) to determine the pigment content. The calculation formula used is as follows:

2 $$\text{Chlorophyll a}\left(mg {g}^{-1}FW\right)={12.21\text{A}}_{663}-{2.81\text{A}}_{645}$$

3 $$\text{Chlorophyll b}\left(mg {g}^{-1}FW\right)={20.13\text{A}}_{645}-{5.03\text{A}}_{663}$$

4 $$\text{Carotenoids}\left(mg {g}^{-1}FW\right)={1000\text{A}}_{470}-{3.27\text{C}}_{a}-{104\text{C}}_{b}/229$$

5 $$\text{Total chorophyll content }\left(mg {g}^{-1}FW\right)=\text{Chl a}+\text{Chl b}$$

#### Determination of gas exchange parameters

The net photosynthetic rate (Pn), stomatal conductance (Gs), and intercellular carbon dioxide concentration of the first fully unfolded leaf of rice plants were measured under various treatments using the portable photosynthetic measurement system Li-6400 (Li-Cor Inc, USA). These measurements were taken between 9:00–11:00 am, seven days after saline-sodic and zinc treatments were applied. For each treatment, 3 rice seedlings with uniform growth were selected for measurement to ensure accuracy. The leaf chamber's temperature was maintained at approximately 26 °C, and the light intensity was set at 800 μmol·m^−2^ s^−1^. Additionally, the CO_2_ concentration was kept at 400 μmol mol^−1^, and the relative humidity ranged between 60 to 70% during the measurements.

#### Chlorophyll a fluorescence transient and 820 nm reflection

After subjecting the rice leaves to 7 days of saline-sodic stress and zinc treatment, their chlorophyll fluorescence was measured. For each treatment, 9 rice seedlings with uniform growth were selected for measurement to ensure accuracy. The leaves with uniform growth in each treatment were dark-adapted for 30 min. An M-PEA fluorometer (Hansatech, UK) was utilized to measure the Chl a fluorescence rise kinetics *OJIP* curve under 1 s pulsed continuous red light (3000 μmol (photons) m^−2^ s^−1^). The fluorescence data were recorded at varying sampling rates: from 0.01 to 0.3 ms, data recorded every 10 μs; from 0.3 to 3 ms, data recorded every 100 μs; from 3 to 30 ms, data recorded every 1 ms; from 30 to 300 ms, data recorded every 10 ms; from 300 to 1000 ms, data recorded every 100 ms [33]. Subsequently, the data were analyzed using the *JIP* test. Fast fluorescence curve *OJIP* calculations were also conducted: *OP* normalized *V*_t_ = (*F*_t_ − *F*_O_)/(*F*_M_ − *F*_O_), *OJ* normalized *W*_OJ_ = (*F*_t_ − *F*_O_)/(*F*_J_ − *F*_O_), *OI* normalized *W*_OI_ = (*F*_t_ − *F*_O_)/(*F*_I_ − *F*_O_). The definitions and calculation formulas for each parameter can be found in Table 2.

**Table 2:** Formulae and explanation the technical data of the *OJIP* curves and the selected *JIP*-test parameters used in this study

Technical fluorescence parameters	
* F*_t_	Fluorescence at time t after onset of actinic illumination
* F*_o_ = *F*_20 µs_	Minimal fluorescence, when all *PSII* RCs are open
* F*_K_≡*F*_300 µs_	Fluorescence intensity at the *K*-step (300 µs) of *OJIP*
* F*_J_≡*F*_2 ms_	Fluorescence intensity at the *J*-step (2 ms) of *OJIP*
* F*_I_≡*F*_30 ms_	Fluorescence intensity at the *I*-step (30 ms) of *OJIP*
* F*_P_ (= *F*_m_)	Maximal recorded fluorescence intensity, at the peak P of OJIP
* V*_t_ = (*F*_t_-*F*_o_)/(*F*_m_-*F*_o_)	Relative variable fluorescence at time t
* W*_OJ_ = (*F*_t_-*F*_o_)/(*F*_J_-*F*_o_)	Ratio of variable fluorescence *F*_t_-*F*_o_ to the amplitude *F*_J_-*F*_o_
* V*_J_ = (*F*_J_-*F*_o_)/(*F*_m_-*F*_o_)	Relative variable fluorescence at the *J*-step
* W*_K_ = (*F*_K_-*F*_o_)/(*F*_J_-*F*_o_)	Relative variable fluorescence at the *K*-step to the amplitude *F*_J_-*F*_o_
**Quantum efficiencies or flux ratios**	
_*φ*__Po_ = TR_o_/ABS = 1-*F*_o_/*F*_m_	Maximum quantum yield for primary photochemistry
_*ψ*__Eo_ = ET_o_/TR_o_ = (1-*V*_J_)	Probability that an electron moves further than *Q*_A_
_*φ*__Eo_ = ET_o_/ABS = (1-*F*_o_/*F*_m_) (1-*V*_J_)	Quantum yield for electron transport (ET)
_*φ*__Do_ = 1-φ_Po_ = *F*_o_/*F*_m_	Quantum yield (at t = 0) of energy dissipation
_*φ*__Ro_ = RE_o_/ABS = *φ*_Po_•*ψ*_E__o_•*φ*_Ro_ = *φ*_Po_•(1-*V*_I_)	Quantum yield for reduction of the end electron acceptors at the *PSI* acceptor side (RE)
_*δ*Ro_ = RE_o_/ET_o_ = (1-*V*_I_)/(1-*V*_J_)	Probability that an electron is transported from the reduced intersystem electron acceptors to the final electron acceptors of *PSI* (RE)
**Specific energy fluxes and Performance indexes**	
M_0_ = 4(*F*_300s_ -*F*_o_)/(*F*_m_-*F*_o_)	Approximated initial slope (in ms) of thefluorescence transient normalized on the maximal variablefluorescence
ABS/RC = M_0_· (1–*V*_J_)· (1–*φ*_Po_)	Absorption flux per RC
TR_o_/RC = M_0_· (1–*V*_J_)	Trapped energy flux per RC (at t = 0)
ET_o_/RC = M_0_· (1–*V*_J_)·*ψ*_Eo_	Electron transport flux per RC (at t = 0)
DI_o_/RC = (ABS/RC) – (TR_o_/RC)	Dissipated energy flux per RC (at t = 0)
RE_o_/RC = M_0_· (1–*V*_J_)·*ψ*_Eo_·*φ*_Ro_	Reduction of end acceptors at *PSI* electron acceptor side per RC (at t = 0)
$PI_{ABS}=(\frac{RC}{ABS})(\frac{\varphi_{po}}{1-\varphi_{po}})(\frac{\Psi_{Eo}}{1-\Psi_{Eo}})$	Performance index on absorption basis
**820 nm modulated reflectance**	
ΔMR_fast_/MR_0_ = (MR_0_-MR_min_)/MR_0_	The amplitudes of the fast phase
ΔMR_slow_/MR_0_ = (MR_max_-MR_min_)/MR_0_	The amplitudes of the slow phase
* V*_ox_ = (MR_0.7 ms_-MR_2 ms_)/1.3	*V*_ox_ is defined as the slope of the fast descending phase (MR_0_ to MR_min_)
* V*_red_ = (MR_9 ms_-MR_30 ms_)/21	*V*_red_ is defined as the slope of the slow ascending phase (MR_min_ to MR_max_)

Subscript “0” (or “o” when written after another subscript) indicates that the parameter refers to the onset of illumination, when all RCs are assumed to be open

Meanwhile using M-PEA (Hansatech, UK), we conducted a red pulse of 3000 μmol (photon) m^−2^ s^−1^ for 2 s to generate a modulated reflection curve at 820 nm and analyzed the rise kinetics. In our measurement setup, we applied a sequence of light pulses including a 1-s red pulse (3000 μmol m^−2^ s^−1^), a 10-s far-red pulse, and another 1-s red pulse (3000 μmol m^−2^ s^−1^) to trigger electron transfer. The reflectance measured around 820 nm corresponds to the redox status of PC^+^ and P700^+^ indicating the activity of *PSI*. The ratio MR/MR_0_, where MR_0_ represents the signal value at the start of the actinic illumination (0.7 ms, the first reliable MR measurement), is used to track the oxidation of *PSI* carriers leading to the formation of PC^+^ and P700^+^ (*V*_ox_), as well as the reduction of PC^+^ and P700^+^ (*V*_red_). [34]. Parameters like MR_fast_/MR_0_, MR_slow_/MR_0_, *V*_OX_, and *V*_red_ are further detailed in Table 2 for reference.

#### Determination of soluble sugars, starches and related metabolic enzymes

After subjecting the rice plants to 7 days of salt, sodium, and zinc treatment, we carefully selected six plants with similar growth for further analysis. To begin, we collected the leaves and roots from three rice seedlings and subjected them to a heat treatment at 105 °C for 30 min. Following this, the samples from each section were dried and weighed at 80 °C. Subsequently, the samples were crushed and passed through a 100-mesh sieve before being saved for testing. We determined the sucrose and fructose content using the resorcinol colorimetric method [35]. Additionally, we determined the starch content in both the rice leaves and roots using anthrone colorimetry [31]. The remaining three rice seedlings' leaves and roots were frozen in liquid nitrogen and stored at -80 °C for enzyme activity analysis. Specifically, we analyzed the activity of invertase (acidic and neutral), sucrose synthetase, sucrose phosphate synthetase, ADP-glucose pyrophosphorylase, and amylase. The enzyme activity analysis was carried out using kits provided by the company (Shanghai Enzyme Linked Biotechnology Co., LTD., China.)

#### Statistical analyses

All data were collected and analyzed using Microsoft Excel 2019 software. Subsequently, the data were analyzed using the SPSS statistical package version 22 (IBM Corp., Armonk, NY, USA). Descriptive statistics were employed to test the mean value and standard error of measurement parameters. One-way analysis of variance (ANOVA) was conducted in this study, and Duncan's multiple comparison method was utilized. The observed differences in comparisons were found to be statistically significant (p < 0.05). The results are presented as standard error (SE). The charts were generated using Origin 2021 software.

## Results

### Plant growth

**Fig. 1:**
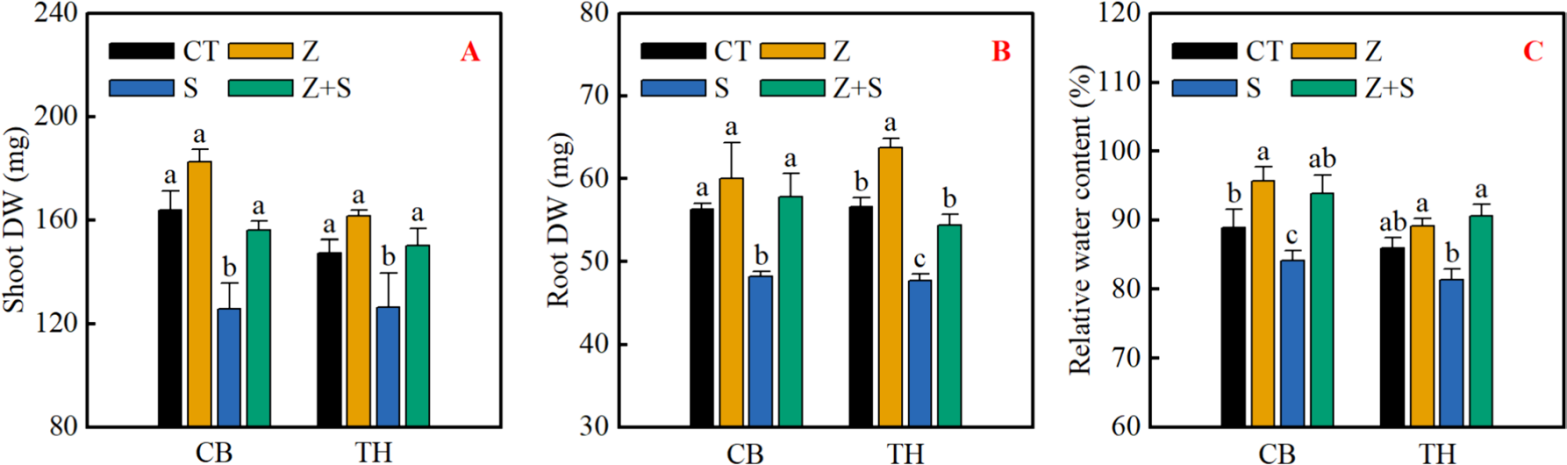
Effect of zinc on dry weight of leaves (**A**) and roots (**B**) and relative water content of leaves (**C**) of rice seedlings under saline-sodic stress. Different letters represent the significant differences between treatments at *p* ≤ 0.05. Values are mean ± SE (*n* = 3). CT: no saline-sodic and no zinc treatment; Z: zinc treatment; S: saline-sodic treatment; Z + S: saline-sodic and zinc treatment. CB: Changbai 9; TH: Tonghe 899

It can be seen from Fig. 1 that under non-stress treatment, adding zinc had no significant effect on the aboveground dry weight of the two varieties, root dry weight and leaf relative water content of 'Changbai 9', but significantly increased the root dry weight and relative water content of 'Tonghe 899'. In comparison to CT treatment, the application of saline-sodic stress resulted in a significant decrease in the above-ground and root dry weight, as well as the relative leaf water content of both rice varieties. Conversely, the addition of zinc had a positive impact on the biomass and relative water content of the two rice varieties.

### Photosynthetic pigment and gas exchange parameters

**Fig. 2:**
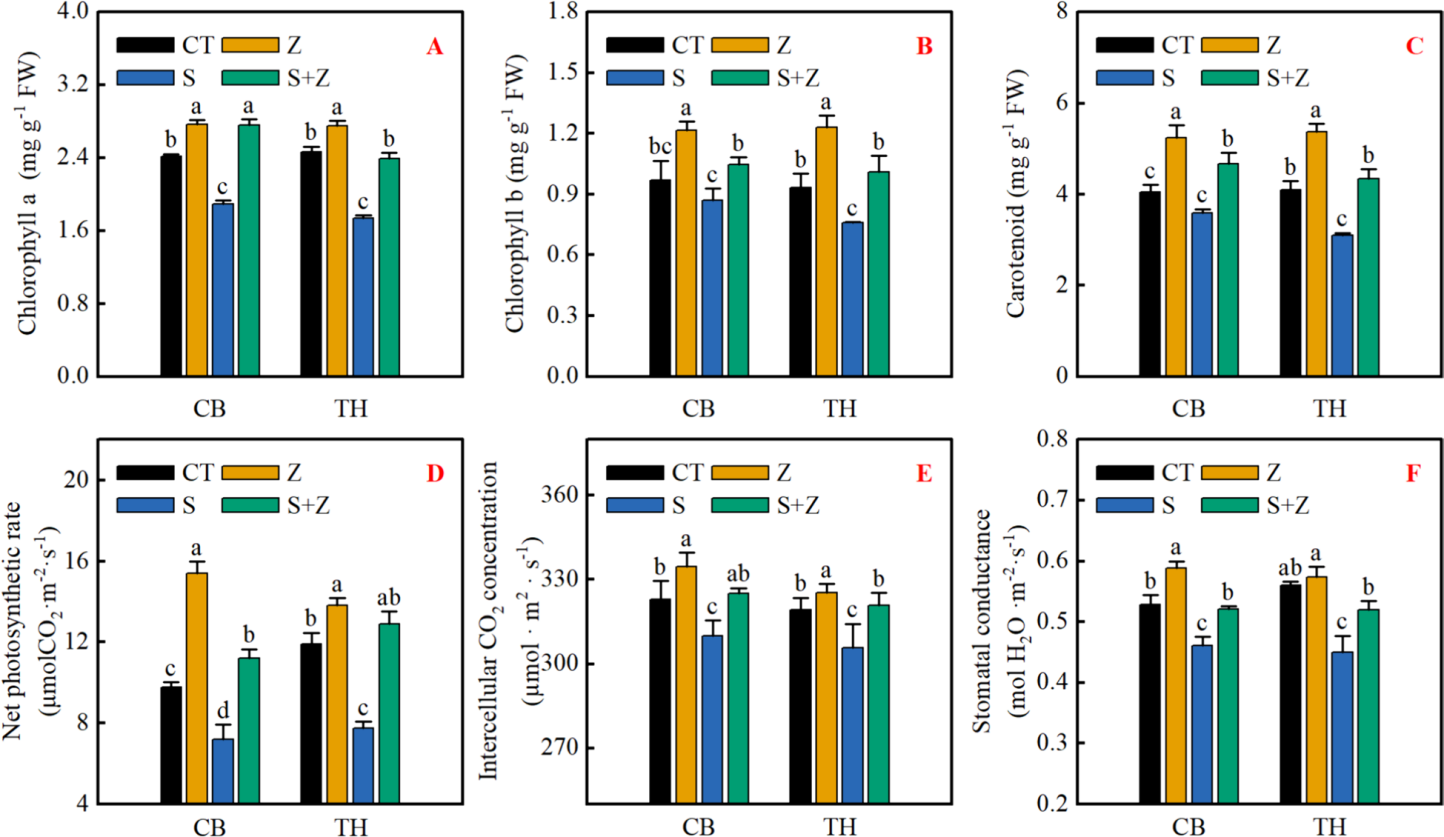
Effect of zinc on chlorophyll a (**A**), chlorophyll b (**B**), and carotenoid (**C**) contents, as well as on the net photosynthetic rate (**D**), intercellular carbon dioxide concentration (**E**), and stomatal conductance (**F**) of rice leaves under conditions of saline-sodic stress. Different letters represent the significant differences between treatments at *p* ≤ 0.05. Values are mean ± SE (*n* = 3). CT: no saline-sodic and no zinc treatment; Z: zinc treatment; S: saline-sodic treatment; Z + S: saline-sodic and zinc treatment. CB: Changbai 9; TH: Tonghe 899

Figure 2 illustrates that in non-stress conditions, the addition of zinc notably enhances chlorophyll, carotenoid content, net photosynthetic rate, intercellular carbon dioxide, and stomatal conductance in 'Changbai 9' of the two varieties. However, the effect of zinc on 'Tonghe 899' There was no significant effect on stomatal conductance. When exposed to saline-sodic stress, both varieties experienced a significant reduction in chlorophyll, carotenoid content, and gas exchange parameters. The external application of zinc can mitigate saline-sodic stress and substantially increase pigment content and gas exchange parameters in rice leaves. Therefore, adding zinc under saline-sodic stress conditions aids in the synthesis of chloroplast pigments in rice leaves, diminishes chlorophyll and carotenoid degradation, and enhances gas exchange parameters.

### The relative variable fluorescence *V*_t_, the *O*—*J* phase and The *O*–*I* phase

**Fig. 3:**
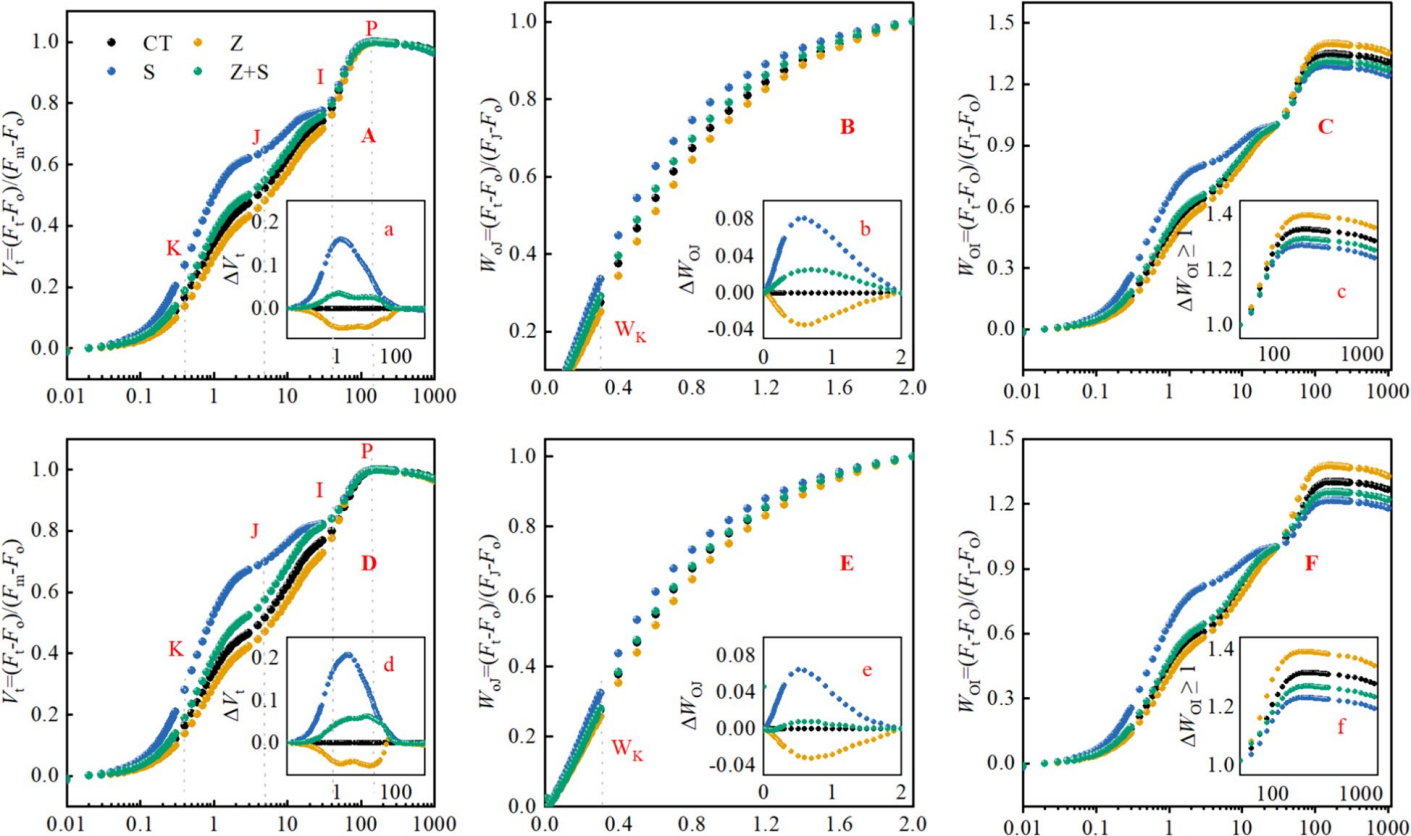
Effect of zinc on the *V*_t_ (**A**, **D**), *O*-*J* (**B**, **E**), and *O*-*I* (**C**, **F**) phases of variable chlorophyll a fluorescence in two rice varieties 'Changbai 9' (**A**,** B**,** C**) and 'Tonghe 899' (**D**,** E**,** F**) under saline-sodic stress. Values are mean ± SE (*n* = 9). CT: no saline-sodic and no zinc treatment; Z: zinc treatment; S: saline-sodic treatment; Z + S: saline-sodic and zinc treatment

Figure 3 (A, D) displays the *OJIP* (chlorophyll fluorescence induction kinetics) curve of rice. In the absence of stress, the *J* and *I* points in the *OJIP* curves of both rice cultivars were reduced by zinc. Conversely, saline-sodic stress heightened the *J* and* I* points in the *OJIP* curves of both rice varieties. However, the application of zinc mitigated the saline-sodic stress and decreased the *J* and *I* points in the *OJIP* curves. When exposed to saline-sodic stress, both varieties displayed a shift in fluorescence rising kinetics from *OJIP* to *OKJIP*. The Δ*V*_t_ (Fig. 3a,d) measurement of 'Tonghe 899' was lower under the same treatment, indicating that it was more susceptible to salinity than 'Changbai 9'. The application of zinc resulted in a decrease of *K*, *J*, and *I* points under saline-sodic stress, with significant changes observed in *K* and *J* points.

The analysis of Fig. 3B,E involves the double standardization of *K*-step *O*-*J* phases. The *K* step, a new intermediate step occurring at 300 μs, is often attributed to limitations in electron transfer on the donor side of *PSII*. This study observed that the *O*-*J* curve of rice leaves treated with saline-sodic was higher than that of leaves treated with saline-sodic and zinc combination. This suggests that saline-sodic treatment impairs the activity of the oxygen extraction complex (*OEC*), while zinc application enhances the activity of the oxygen extraction complex (*OEC*). Furthermore, the 'Tonghe 899' variety exhibited a more pronounced effect compared to the 'Changbai 9' variety (Fig. 3B,E). Phase *O*-*I* analysis revealed a consistent trend between the two rice varieties, as depicted in Fig. 3C,F. The amplitude of the *I*-*P* phase in the *W*_OI_ ≥ 1 component indicates the magnitude of the terminal electron acceptor pool on the *PSI* acceptor side. A smaller amplitude signifies a smaller terminal electron acceptor pool on the *PSI* acceptor side. The findings demonstrated that saline-sodic stress reduced the size of the receptor pool, whereas exogenous zinc increased its size.

### The *JIP* parameters estimating the quantum yields, efficiencies and Probabilities

**Fig. 4:**
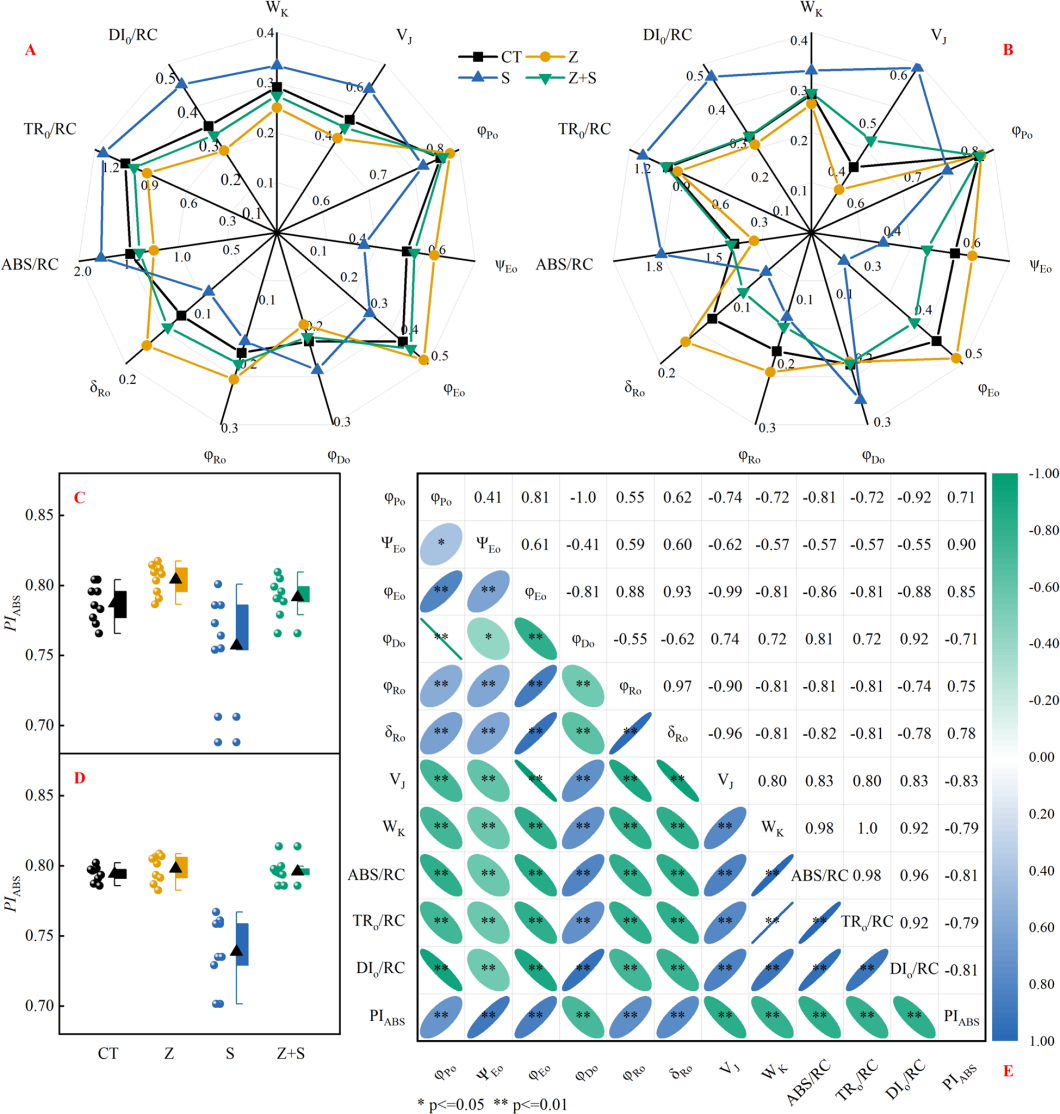
Effects of zinc on quantum yield and specific energy flux of *PSII* reaction center of two rice varieties 'Changbai 9' (**A**) and 'Tonghe 899' (**B**), and energy conservation performance index (**C**, **D**) of *PSII* in two rice varieties (Changbai 9, Tonghe 899) under saline-sodic stress. Fig. **E** presents the correlation analysis of *PI*_ABS_, *V*_*J*_, *W*_K_, *φ*_Do_, *φ*_Po_, *ψ*_Eo_, *φ*_Eo_, *φ*_Ro_, *δ*_Ro_, ABS/RC, DI_0_/RC and TR_0_/RC. * indicates significant correlation at *P* < 0.05, ** indicates significant correlation at *P* < 0.01. *φ*_Po_, maximum quantum yield for primary photochemistry; *ψ*_Eo_, probability that an electron moves further than *Q*_A_; *φ*_Eo_, quantum yield for electron transport (ET); *φ*_Do_, quantum yield (at t = 0) of energy dissipation; *φ*_Do_, quantum yield (at t = 0) of energy dissipation; *φ*_Ro_, quantum yield for reduction of the end electron acceptors at the PSI acceptor side (RE); *δ*_Ro_, probability that an electron is transported from the reduced intersystem electron acceptors to the final electron acceptors of *PSI* (RE); ABS/RC, Absorbed photon flux per active *PSII*; TR_0_/RC, Trapped energy flux per active *PSII*; DI_0_/RC, Dissipated energy (as heat and fluorescence) flux per active *PSII*; ET_0_/RC, Electron flux from *Q*_A_^‒^ to the PQ pool per active *PSII*; RE_0_/RC, Electron flux from *Q*_A_^‒^ to the final electron acceptors of PSI per active *PSII*. Values are mean ± SE (*n* = 9). CT: no saline-sodic and no zinc treatment; Z: zinc treatment; S: saline-sodic treatment; Z + S: saline-sodic and zinc treatment

To assess the level of *K*-step variation in the *OJIP* curve, we determined the normalized relative variable fluorescence (*W*_K_) of *K*-step. As depicted in Fig. 4A,B, the *W*_K_ value of 'Changbai 9' and 'Tonghe 899' decreased by 17.82% and 13.66%, when subjected to saline-sodic and zinc treatment compared to saline-sodic treatment. Additionally, *V*_J_ values were calculated to evaluate the connectivity and receptor-side properties of *PSII* components. Saline-sodic and zinc treatment resulted in decreased *V*_J_ values. In comparison to the S treatment, the *V*_J_ values of 'Changbai 9' and 'Tonghe 899' showed a significant increase of 26.27% and 23.45% respectively under the Z + S treatment.

We also assessed the quantum yield and various relevant parameters of rice seedlings exposed to saline-sodic stress. The results depicted in Fig. 4A, B indicate that under saline-sodic stress conditions, the energy dissipation quantum yield (t = 0) *φ*_Do_ increases, while the quantum yield per unit ABS (*φ*_Po_) decreases. Furthermore, the introduction of zinc leads to an enhancement in the quantum yield values of *φ*_Po_, *φ*_Eo_, *φ*_Ro_, *δ*_Ro_, and *ψ*_Eo_ per unit ABS, along with a reduction in the energy dissipation quantum yield *φ*_Do_. The results presented in Fig. 4C,D demonstrate that the *PI*_ABS_ index of the two rice varieties increased significantly with the addition of zinc when no stress was present. However, saline-sodic stress led to a notable reduction in the *PI*_ABS_ index of both varieties. Fortunately, the application of zinc helped in restoring the *PI*_ABS_ activity index in both varieties.

Under saline-sodic stress conditions, ABS/RC, DI_0_/RC, and TR_0_/RC increased (Fig. 4A,B), indicating that the unit reaction center absorbed more energy primarily for capture and heat dissipation, with less energy being transmitted downstream. Interestingly, the introduction of zinc led to a significant decrease in ABS/RC, DI_0_/RC, and TR_0_/RC, resulting in a more balanced energy flux. The positive impact of zinc on energy absorption, transfer, and transmission in the reaction center under saline-sodic stress is evident.

According to the findings presented in Fig. 4E, there is a positive correlation between *PI*_ABS_ and *φ*_Po_, *ψ*_Eo_, *φ*_Eo_, *φ*_Ro_, and *δ*_Ro_. On the other hand, *PI*_ABS_ is negatively correlated with *V*_J_, *W*_K_, *φ*_Do_, ABS/RC, DI_0_/RC, and TR_0_/RC. Therefore, zinc effectively enhances the connectivity and receptor-side properties of *PSII* components, which is beneficial for improving the quantum yield and efficiency of rice under saline-sodic stress. This results in a reduction of the energy dissipation ratio and improvement in photosynthetic fluorescence performance indicators.

### The reflection at 820 nm

**Fig. 5:**
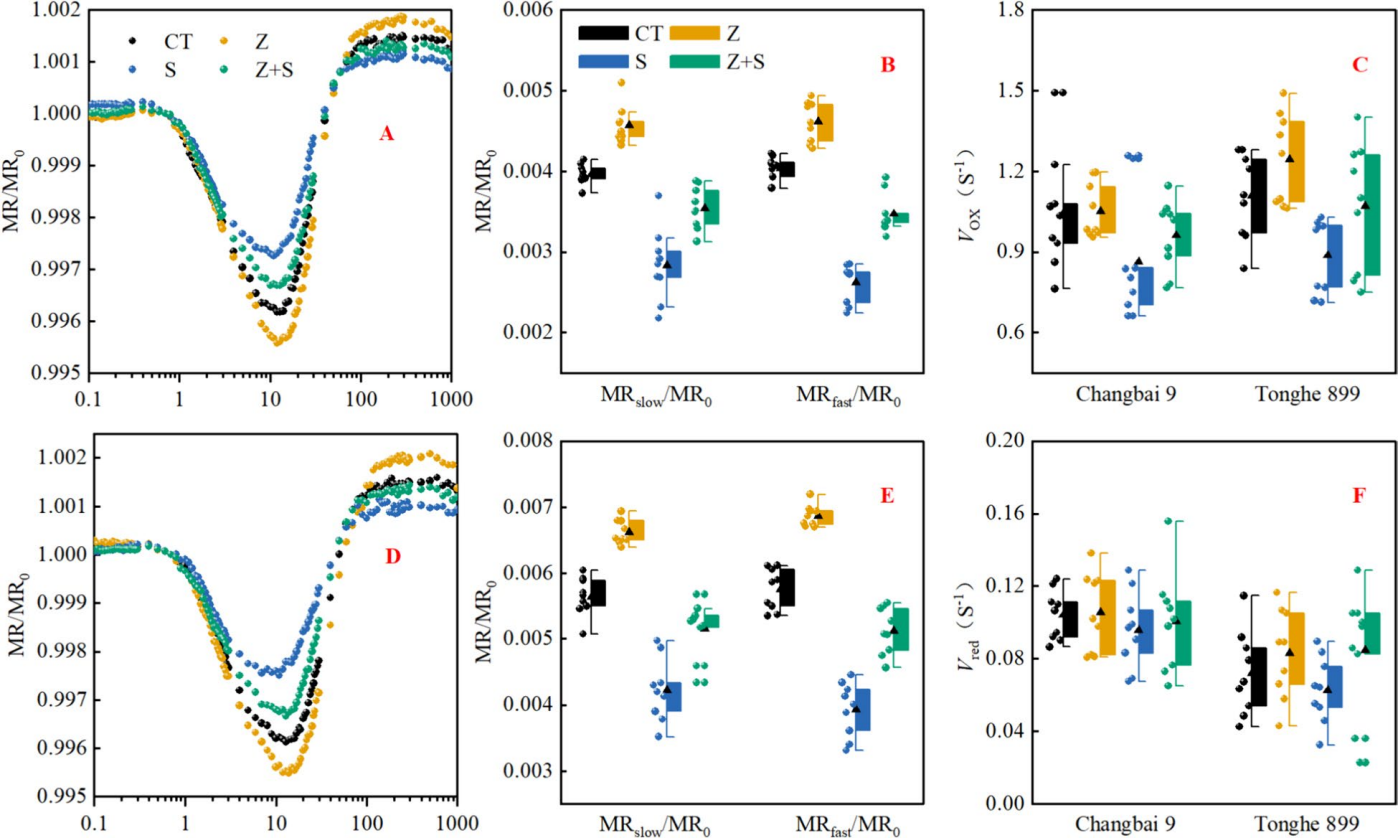
Normalized modulated 820 nm reflectance kinetics (MR_0_ = MR_0.7 ms_) of zinc on 'Changbai 9' rice variety(**A, B**) and 'Tonghe 899' rice variety(**D, E**) under saline-sodic stress. (**B, E**) the amplitudes of the fast phase (ΔMR_fast_/MR_0_) and the slow phase (ΔMR_slow_/MR_0_). (**C, F**) *V*ox, the slope of the fast descending phase (MR_0_ to MR_min_) and *V*red, the slope of the slow ascending phase (MR_min_ to MR_max_). Values are mean ± SE (n = 9). CT: no saline-sodic and no zinc treatment; Z: zinc treatment; S: saline-sodic treatment; Z + S: saline-sodic and zinc treatment. CB: Changbai 9; TH: Tonghe 899

MR/MR_0_ values were determined using 820 nm red light reflection technology and double standardization. In this study (Fig. 5A,D), it was observed that exogenous zinc supplementation reduced the minimum MR/MR_0_ values of the two rice varieties under non-stress treatment. The minimum MR/MR_0_ value of the rice varieties was higher than that of the control group (CT) under saline-sodic stress, but zinc application decreased the minimum MR/MR_0_ value. This indicates that saline-sodic stress slow down the oxidation rate of P700 and PC, while the addition of foreign zinc accelerates the oxidation rate of P700 and PC. ΔMR_fast_/MR_0_ and ΔMR_slow_/MR_0_ represent the amplitude of variation from MR_0_ to MR_min_ and MR_min_ to MR_max_(Fig. 5B,E), respectively, for the determination of *V*_ox_ and *V*_red_. *V*_ox_ and *V*_red_ are defined as the slopes of two different phases in a curve. *V*_ox_ represents the slope from MR_0_ to the rapid descent stage of MR_min_, and *V*_red_ represents the slope from the slow ascent stage of MR_min_ to MR_max_. The results of Fig. 5C,F show that, there was no significant change in the *V*_OX_ and *V*_red_ values of the two rice varieties after adding zinc under no stress condition. Under saline-sodic stress, the *V*_OX_ and *V*_red_ values of the two rice varieties decreased significantly. However, the addition of zinc exogenously led to a significant increase in *V*_OX_ and *V*_red_ values even under saline-sodic stress. The results demonstrated that saline-sodic stress inhibited the rates of oxidation and reduction of P700 and PC. However, the application of zinc alleviated the reduction in the rates of reoxidation and reduction of P700 and PC.

### The contents of carbohydrate

**Fig. 6:**
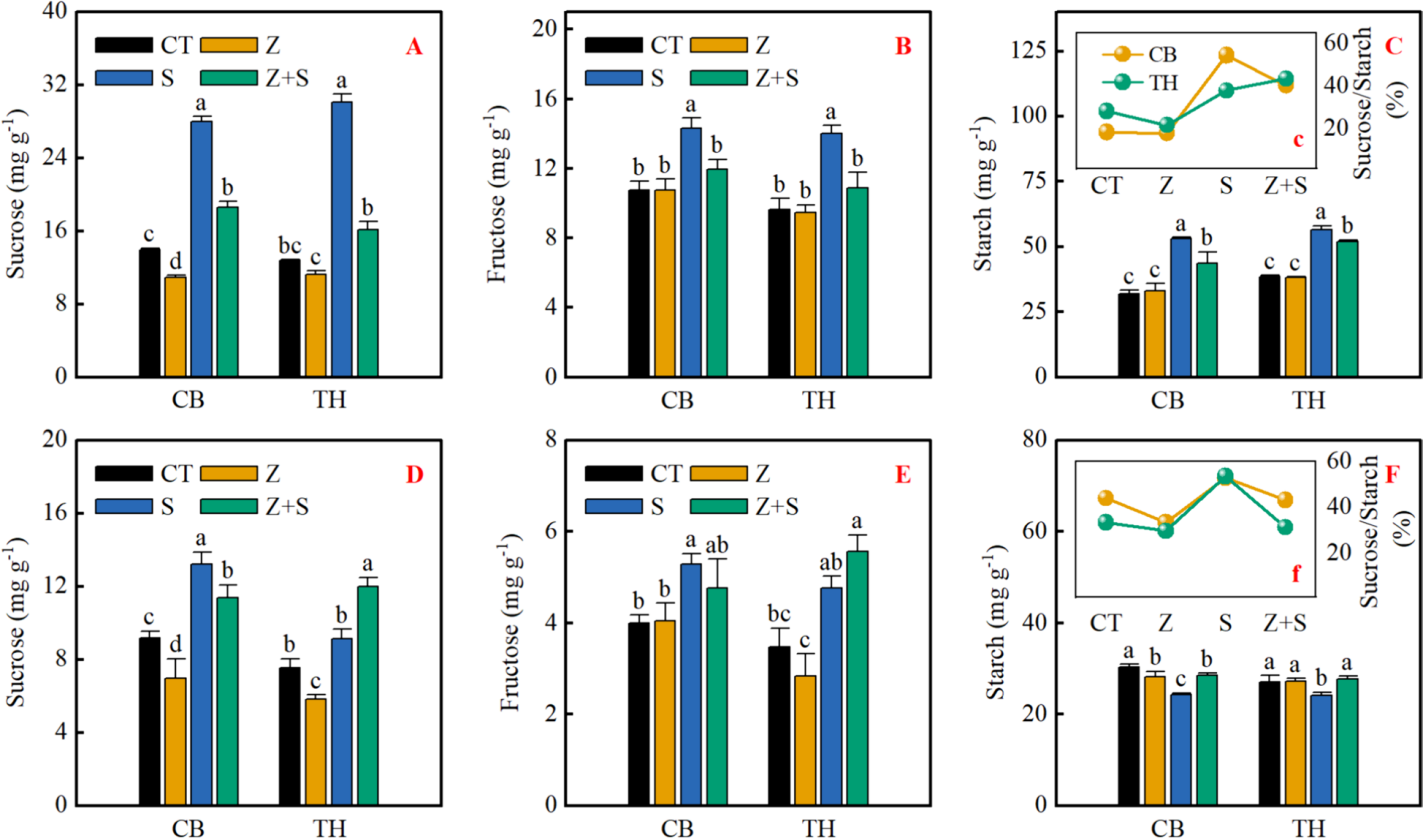
Effects of zinc on sucrose (**A**, **D**), fructose (**B**, **E**) and starch contents (**C**, **F**) and ratio of sucrose to starch contents (**c**, **f**) in rice leaves (**A**, **B**, **C**, **c**) and roots (**D**, **E**, **F**, **f**) under saline-sodic stress. Different letters represent the significant differences between treatments at *p* ≤ 0.05. Values are mean ± SE (*n* = 3). CT: no saline-sodic and no zinc treatment; Z: zinc treatment; S: saline-sodic treatment; Z + S: saline-sodic and zinc treatment. CB: Changbai 9; TH: Tonghe 899

The results presented in Fig. 6 indicate that zinc did not have a significant impact on fructose and starch levels in the leaves and roots of the two varieties under normal conditions. However, saline-sodic stress did lead to significant changes in sucrose, fructose, and starch contents in the leaves of both varieties. Interestingly, the addition of zinc helped alleviate this effect, resulting in a decrease in sucrose, fructose, and starch levels. Specifically, compared to the S + Z treatment, the sucrose content of 'Changbai 9' and 'Tonghe 899' decreased by 33.7% and 46.4%, respectively. Furthermore, under the S + Z treatment, the starch content of 'Changbai 9' and 'Tonghe 899' decreased by 17.7% and 8.2%, respectively. It is worth noting that the ratio of sucrose to starch in both varieties increased under saline-sodic stress, but zinc application significantly reduced this proportion. Furthermore under saline-sodic stress, there was a significant increase in the content of sucrose and fructose in the roots of the two rice varieties, while the starch content decreased. The addition of zinc resulted in a decrease in sucrose and fructose content and the sucrose to starch ratio in the root of 'Changbai 9'. However, zinc led to a significant increase in the starch content of the root system of 'Changbai 9'. In addition, the root systems of 'Tonghe 899' responded differently to zinc, with an increase in sucrose and fructose content and the sucrose to starch ratio, but a significant decrease in starch content for 'Tonghe 899'. Comparatively, the addition of zinc (S + Z) increased the starch content of 'Changbai 9' and 'Tonghe 899' by 17.6% and 14.2% respectively, when compared to the S treatment.

### The carbohydrate metabolism enzyme activity

**Fig. 7:**
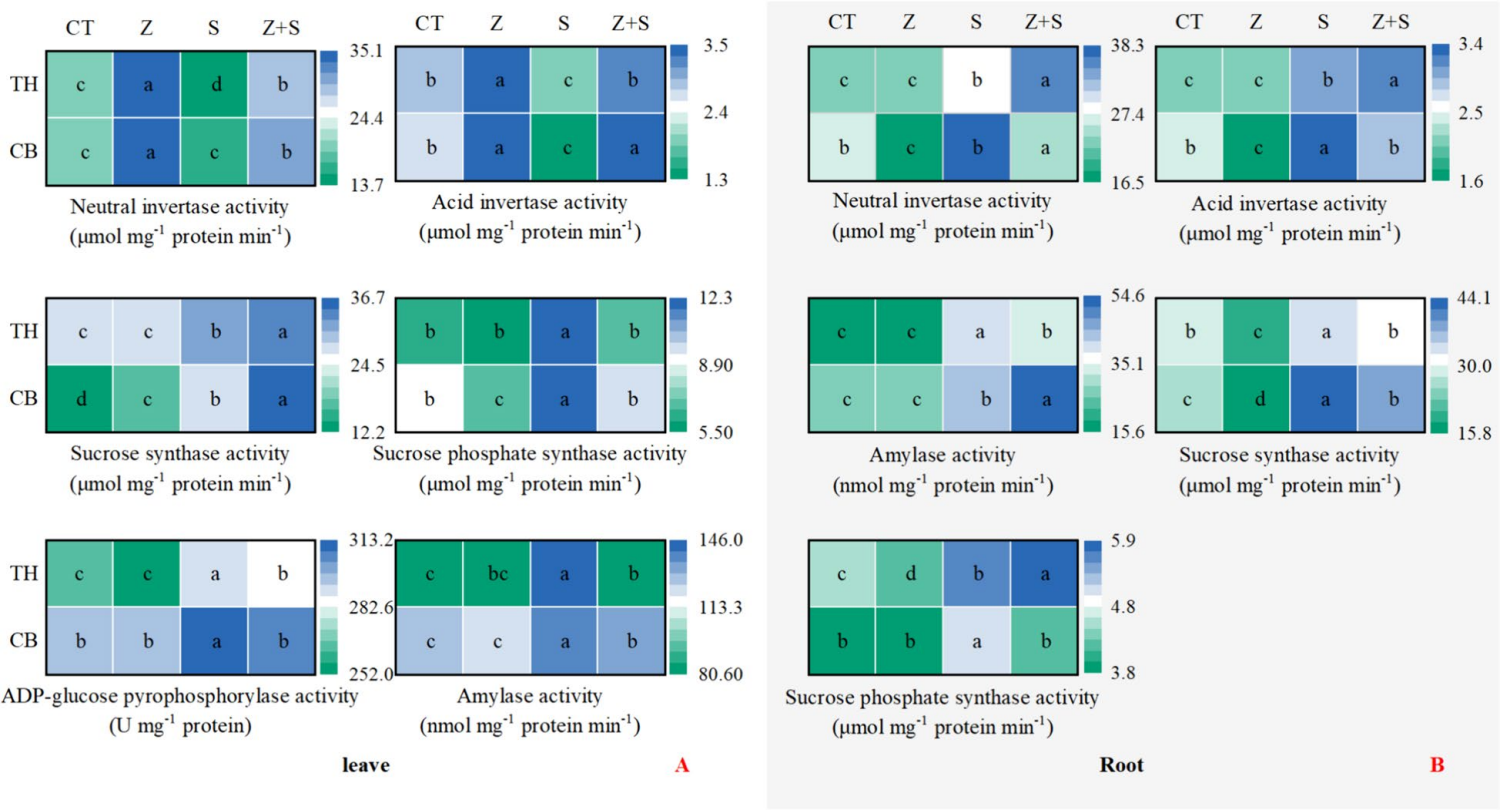
Effect of zinc on carbohydrate metabolism enzyme activity of rice leaves (**A**) and roots (**B**) under saline-sodic stress. Different letters represent the significant differences between treatments at *p* ≤ 0.05. Values are mean ± SE (*n* = 3). CT: no saline-sodic and no zinc treatment; Z: zinc treatment; S: saline-sodic treatment; Z + S: saline-sodic and zinc treatment. CB: Changbai 9; TH: Tonghe 899

Figure 7A shows the effect of zinc on carbohydrate-metabolizing enzymes in the leaves of two rice varieties. Sucrose phosphate synthase is the primary enzyme responsible for catalyzing sucrose synthesis within the cytoplasm. Conversely, sucrose degradation is predominantly facilitated by sucrose synthase and invertase. While sucrose synthase has the ability to both synthesize and break down sucrose, it typically functions primarily in the decomposition of sucrose. ADP-glucose pyrophosphorylase and amylase are crucial enzymes involved in starch synthesis and degradation, respectively. It is evident that the invertase activity of both varieties was significantly increased with zinc supplementation under non-stress treatment. However, the sucrose phosphate synthetase activity was observed to decrease. When subjected to saline-sodic stress, the activities of sucrose synthetase, sucrose phosphate synthetase, ADP-glucose pyrophosphorylase, and amylase in the leaves of both cultivars were notably higher compared to CT. However, the acid invertase activity of 'Changbai 9' and the neutral invertase activity of 'Tonghe 899' were observed to decrease. The invertase activities of both cultivars subjected to saline-sodic stress were significantly increased with the addition of zinc. However, the activities of sucrose phosphate synthetase, ADP-glucose pyrophosphorylase, and amylase were decreased.

Figure 7B shows the effect of zinc on carbohydrate-metabolizing enzymes in the roots of two rice varieties. the addition of zinc from an external source considerably decreased the activity of sucrose synthase in both rice varieties when not subjected to stress. when exposed to saline-sodic stress, the roots of the two rice varieties exhibited a significant increase in the activities of invertase, sucrose synthetase, sucrose phosphate synthetase, and amylase. When exposed to saline-sodic stress, the addition of zinc from an external source led to a significant increase in the activities of starch synthetase in the root system of 'Changbai 9'. However, the activities of invertase, sucrose synthetase, and sucrose phosphate synthetase decreased. Meanwhile, the addition of zinc also resulted in an increase in the activities of invertase and sucrose phosphate synthetase, but a decrease in the activities of sucrose synthetase and amylase in the root system of 'Tonghe 899'.

## Discussion

### Effects of zinc on growth, chlorophyll content and gas exchange parameters of rice under saline-sodic stress

Biomass serves as a reliable indicator of how plants react to abiotic stress environments, such as salinity [36]. Various abiotic stresses, including salinity, can impede crop growth by restricting photosynthesis [37–39]. Research indicates that saline-sodic stress leverages light energy to trigger the excessive production of reactive oxygen species, leading to chlorophyll breakdown and further constraining photosynthesis and rice development [40]. Additionally, saline-sodic stress disrupts rice photosynthesis by compromising the integrity of the plasma membrane [41]. Consistent with previous findings, our study demonstrates that saline-sodic stress significantly impairs net photosynthesis, stomatal conductance, and intercellular carbon dioxide concentration in rice leaves (Fig. 2D,E,F). The application of zinc has been shown to mitigate saline-sodic stress, resulting in a notable improvement in the aforementioned parameters of rice leaves (Fig. 2). This highlights the significance of zinc in enhancing rice resilience under saline-sodic stress. Zinc not only alleviates saline-sodic stress but also enhances the integrity of the plasma membrane [41]. It also helps maintain high concentrations of potassium in protective cells, improves stomatal conductance of rice leaves, and promotes photosynthesis of rice in saline-sodic land [42]. Additionally, zinc, as a vital component of chloroplasts, plays a key role in the formation and function of chlorophyll [43]. Our study further validates these findings, showing that zinc application benefits pigment synthesis in rice leaves under saline-sodic stress (Fig. 2A,B,C), facilitating rice photosynthesis. Consequently, zinc can enhance rice's tolerance to saline-sodic stress, improve photosynthesis, and ultimately promote rice growth (Fig. 1).

### Effects of zinc on chlorophyll fluorescence in rice under saline-sodic stress

Chlorophyll fluorescence is a widely used method to assess plant photosynthetic performance [44, 45]. This research employed rapid chlorophyll fluorescence kinetics technology and *JIP*-text analysis to investigate the impact of zinc on chlorophyll fluorescence in two rice varieties under saline-sodic stress conditions. Previous studies have indicated that saline-sodic stress leads to a decrease in the efficiency of the PSII reaction center and significant changes in chlorophyll fluorescence parameters [25], aligning with the findings of this study. Figure 3A,B illustrates that the *J*-step and* I*-step in the *OJIP* curves of both rice varieties increased under saline-sodic stress. However, the application of zinc in this study resulted in a reduction of both *J*-step and *I*-step in the *OJIP* standard curve for both varieties, with a more pronounced change observed in the *J*-step. This may be that under saline-sodic stress, the destruction of the *PSII* reaction center *D*_1_ protein may lead to a decrease in electron flow from the plastoquinone pool of *PSII* and an imbalance in the reoxidation of the plastoquinone pool due to *PSI* activity. Studies have shown that zinc plays a crucial role in energy transport, protein synthesis, protecting protein structure, and maintaining cell membrane integrity [46]. Therefore, exogenous zinc application has been found to repair damage caused by saline-sodic stress to the D_1_ protein, facilitating electron transfer from *Q*_A_ to the secondary quinone receptor* Q*_B_ and aiding in the maintenance of quinone pool reduction equilibrium between the two photosystems [47]. Additionally, Li et al. discovered that the degradation of *PSII* protein under saline-sodic stress conditions is a consequence of excessive reactive oxygen species accumulation, particularly under saline-sodic stress [48]. However, the application of zinc has been shown to alleviate saline-sodic stress, decrease reactive oxygen species accumulation [41], and reduce *PSII* protein degradation, leading to improved chlorophyll fluorescence in rice leaves. Furthermore, this study observed a distinct intermediate '*K*' step at around 300 ms under saline-sodic stress in both varieties, with the 'Tonghe 899' saline-sodic tolerant variety exhibiting a more pronounced magnitude of this step. Research indicates that the emergence of the '*K*' step may be attributed to the delay in Oxygen-Evolving Complex (*OEC*) transferring electrons to oxidized chlorophyll during saline-sodic stress, leading to an electron transfer imbalance between *PSII* donors and acceptors. However, the introduction of external zinc has been observed to diminish the '*K*' step, suggesting that zinc has the potential to mitigate saline-sodic stress, safeguard the *OEC*, and reinstate the equilibrium in electron transfer between *PSII* donors and acceptors.

*PI*_ABS_ serves as a valuable standard for evaluating crop stress damage due to its high sensitivity and ability to detect various stress conditions [49]. The study demonstrated that saline-sodic led to a decrease in *PI*_ABS_ content in rice leaves, while zinc treatment increased it. *PI*_ABS_ composed of three components: RC/ABS, *φ*_Po_, and *ψ*_Eo_ [18]. Consistent with Demetriou findings, our study revealed a significant positive correlation between *PI*_ABS_ and *φ*_Po_ and *ψ*_Eo_, as well as a significant negative correlation with ABS/RC (Fig. 4). Figure 4A,B illustrated that both rice varieties exhibited a decrease in *φ*_Po_ and an increase in *φ*_Do_ under saline-sodic stress. Li similarly observed a decrease in photochemical reaction efficiency (*φ*_Po_) under saline-sodic stress conditions. This decrease resulted in an increase in energy dissipation, including heat, fluorescence, and energy transfer to other systems [50]. The application of zinc reduces energy dissipation, increases *φ*_Po_, and decreases *φ*_Do_. This could be attributed to zinc's role in the electron transport chain, where higher zinc levels improve electron acceptor efficiency and facilitate electron transport between *PSI* and *PSII* [51, 52]. Under zinc deficiency conditions, energy is dissipated as heat, leading to *φ*_Do_ production. Furthermore, *ψ*_Eo_ reflects the likelihood of electrons moving beyond *Q*_A_^−^. Our study revealed that saline-sodic stress decreased the excitation pressure of *PSII* (*ψ*_Eo_), whereas zinc increased it. Furthermore, *PSII* exhibited similar changes in quantum yields for *ψ*_Eo_ and *φ*_Eo_, as illustrated in Fig. 4D. These alterations could be associated with variations in the *V*_J_ (Fig. 4A,B). It's worth noting that *V*_J_ has a significant negative correlation with *ψ*_Eo_ and *φ*_Eo_. An increase in *V*_J_ was linked to damage on both sides of the *PSII* donor and recipient under saline-sodic stress. And the rise in *V*_J_ was attributed to a decrease in *Q*_A_ and plastoquinone (PQ) [45]. Moreover, Fig. 4A,B illustrates that reductions in *φ*_Po_, *φ*_Eo_, and *φ*_Ro_ under saline-sodic stress suggest that absorbed light energy is primarily utilized for capture (TR_0_/RC) and dissipation (DI_0_/RC), resulting in a decline in the probability of *PSI* final electron acceptor (*δ*_Ro_). Exogenous zinc application can enhance electron acceptor efficiency in the electron transport chain, facilitating electron transfer between *PSI* and *PSII*, thereby boosting the likelihood of electron transfer and *PSI* final electron acceptor (*δ*_Ro_).

The kinetics of photoinduced 820 nm reflection (MR/MR_0_) can be utilized to identify the buildup of P700 in the PSI reaction center [30], as well as the subsequent rereduction of PC^+^and P700^+^ by electrons that were initially captured by P680 [53]. This study demonstrated that saline-sodic stress significantly impacted the oxidation–reduction rate of PC and P700, whereas the addition of external zinc enhanced the oxidation–reduction rate of PC and P700 (Fig. 5A,D). Additionally, to further assess the redox rates of PC and P700 using the MR_820_ signal, we calculated the values of ΔMR_fast_/MR_0_ and ΔMR_slow_/MR_0_ (Fig. 5B,E) as well as the values of *V*_ox_ and *V*_red_ (Fig. 5C,F). The study revealed that saline-sodic stress decreased the ratios of △MR_fast_/MR_0_ and △MR_slow_/MR_0_, along with the values of *V*_ox_ and *V*_red_. Conversely, the application of zinc led to an increase in the ratios of △MR_fast_/MR_0_ and △MR_slow_/MR_0_, as well as the values of *V*_ox_ and *V*_red_. These findings suggest that zinc supplementation positively impacts the oxidation of PC and P700, or the re-reduction of PC^+^ and P700^+^. This effect may be attributed to zinc's ability to enhance electron flow through PSI, thereby accelerating the redox rates of PC and P700 (Fig. 3C,F), the maximum amplitude of *W*_OI_ ≥ 1 is indicative of the terminal electron acceptor pool size on the *PSI* receptor side. Zinc administration has been shown to increase the size of this pool. Additionally, the study also show that exogenous zinc application enhances electron acceptor efficiency (*δ*_Ro_) in the electron transport chain (Fig. 4A,B). This improvement facilitates electron transfer between *PSI* and *PSII*, leading to enhanced efficiency of PC and P700 oxidation–reduction rates.

**Fig. 8:**
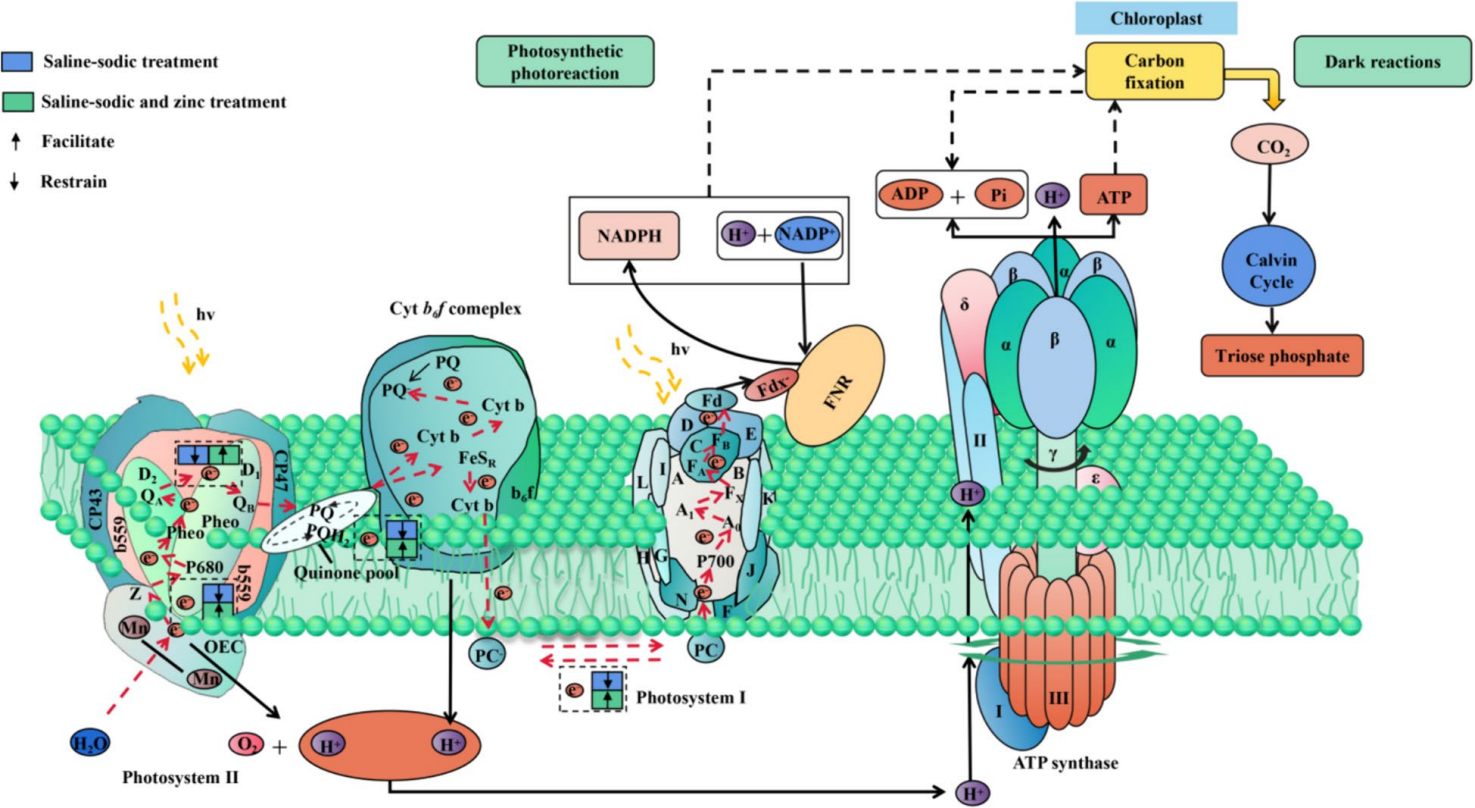
Schematic diagram illustrating the composition of Z-shaped electron transport membrane proteins in the photosynthetic electron transport chain. The protein complex includes *PSII*, cytochrome b6f complex, *PSI*, and *AT*P synthase. Electrons are initially released from water by the oxygen-releasing complex (*OEC*), then transferred to quinone molecules *Q*_A_ and *Q*_B_, and further to *PSI* via quinone and cyanin. Ultimately, these electrons are utilized for *ATP* synthesis facilitated by *ATP* synthase. Under saline-sodic stress, the *OEC* is significantly impaired, leading to inhibition of the electron acceptor and electron donor of *PSII* and *PSI*. Nonetheless, the addition of zinc supplement mitigates the saline-sodic stress, diminishes the damage to the *OEC*, enhances the electron acceptor and electron donor of *PSII* and *PSI*, and facilitates electron transfer. Consequently, the photosynthetic efficiency of rice leaves in saline-sodic soil is enhanced. Fdx, Ferredoxin. FNR, Ferredoxin *NADP*^+^ reductase

As shown in Fig. 8, saline-sodic stress negatively impacts chlorophyll synthesis and the electron transport chain between *PSI* and *PSII* in rice leaves. Zinc application mitigates damage to the oxygen release complex from saline-sodic stress, enhances performance of *PSII* donor/acceptor sides and the redox rate of PSI, repairs the photosynthetic electron transport chain, and improves the transfer of *Q*_A_ to *Q*_B_. These effects contribute to a more balanced energy distribution, ultimately promoting photosynthesis in rice leaves in saline-sodic soil rice areas.

### Effects of zinc on carbohydrate metabolism in rice under saline-sodic stress

**Fig. 9:**
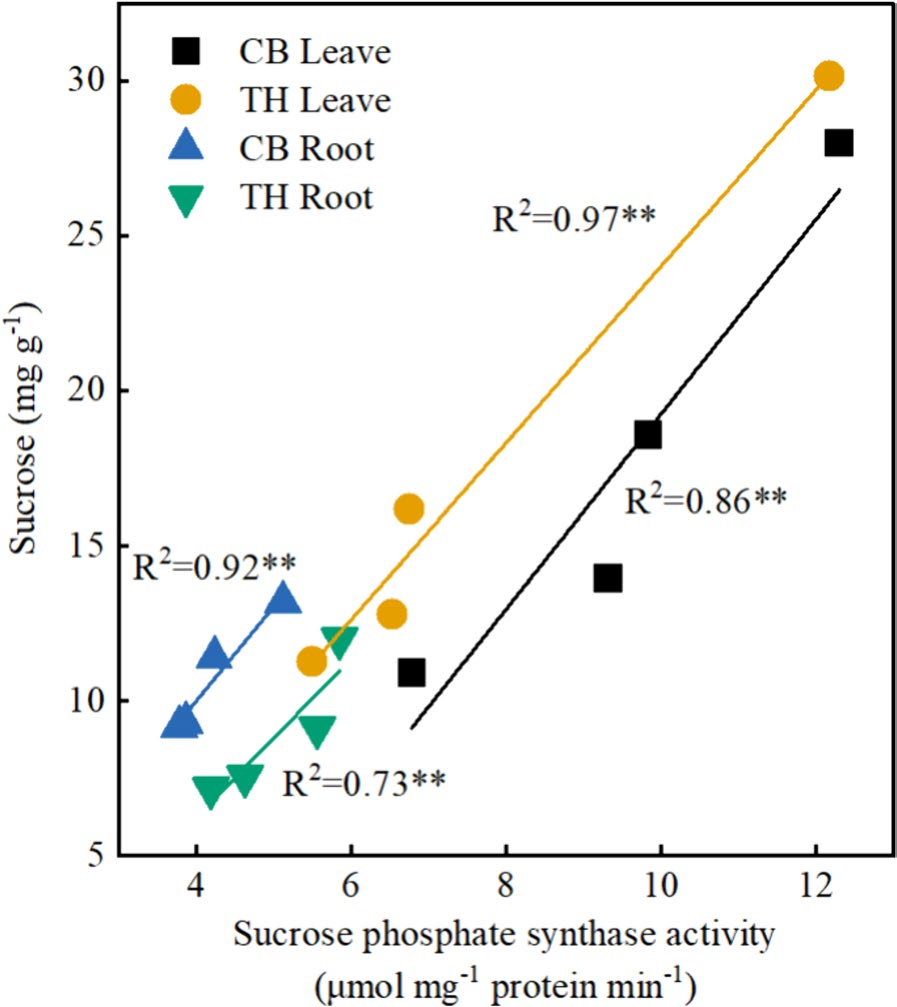
Correlation between sucrose content in rice leaves and roots and sucrose phosphate synthase activity.**is significantly correlated with *P* < 0.01

Photosynthesis is a process that supplies plants with a sufficient carbon source, which in turn promotes the growth and development of crops. However, under saline-sodic stress, plants experience significant inhibition in their ability to absorb and utilize light energy, resulting in impaired photosynthetic capacity [54]. This study discovered that zinc did not have a significant impact on the sucrose, fructose, and starch levels in rice leaves under non-stress conditions. However, when exposed to saline-sodic stress, there was a notable increase in the carbohydrate content in the leaves. Saline-sodic stress impacts the production, transport, distribution, and utilization of sucrose, leading to the buildup of soluble sugars and starches in the source leaves, aligning with Richter's previous research [54]. The application of zinc was found to alleviate these effects by decreasing the levels of sucrose, fructose, and starch in the leaves. This, in conjunction with zinc, could mitigate saline-sodic stress, lower the presence of reactive oxygen species in the source leaves, and enhance the generation of triose phosphate during photosynthesis, thereby facilitating the carbon cycle. On the other hand, sucrose phosphate synthetase serves as the primary enzyme responsible for catalyzing sucrose synthesis in the plant cytoplasm. Additionally, ADP-glucose pyrophosphorylase and amylase are crucial enzymes that play a key role in starch synthesis and degradation, respectively [55]. In this study, in the absence of stress, the addition of exogenous zinc led to a notable increase in invertase activity, while sucrose phosphate synthase activity experienced a significant decrease. Under saline-sodic stress, sucrose phosphate synthetase activity, which promotes sucrose synthesis, increases, while the activity of acidic invertase and neutral invertase, which promote sucrose decomposition, decreases. It is important to note that sucrose synthase activity, which catalyzes sucrose decomposition, increases under saline-sodic stress. Despite this increase, the sucrose content in the leaves did not decrease, possibly because sucrose is being synthesized at a faster rate than it is being broken down. As shown in Fig. 9, sucrose content was significantly positively correlated with sucrose phosphate synthase activity, further supporting this view. Simultaneously, the study revealed that rice leaves experienced a significant increase in starch content under saline-sodic stress, while the application of zinc resulted in a decrease in starch content. This decrease was attributed to the reduced activity of ADP-glucose pyrophosphorylase. Furthermore, the introduction of zinc led to a notable decrease in the sucrose/starch ratio in rice leaves when subjected to saline-sodic stress. This suggests that zinc supplementation could enhance the conversion of sucrose into starch within rice leaves, ultimately aiding in mitigating the transport disruption of photocontracted products caused by the saline-sodic stress [56].

To ensure normal growth in the presence of saline-sodic stress, plants must effectively regulate the transportation and distribution of carbohydrates, as well as their utilization in the reservoir organs [56]. In the context of saline-sodic stress, the roots of plants are the first and most significantly impacted components [57]. This stressor affects the size of the carbohydrate pool by disrupting the transport and distribution of these important nutrients [56]. The study determined that zinc does not have a substantial impact on the levels of sucrose, fructose, and starch in rice roots under normal conditions. However, Saline-sodic stress had a notable impact on the sucrose content in the root system of 'Changbai 9', leading to an increase. However, the application of zinc decreased the sucrose content, possibly due to the rise in invertase activity and reduction in sucrose phosphate synthetase activity. Although sucrose synthetase activity decreased, the accumulation of sucrose indicated that sucrose decomposition exceeded sucrose synthesis. The sucrose content of 'Tonghe 899' root was found to increase significantly under saline-sodic stress, and this was further augmented by the application of zinc. In this study, saline-sodic stress was found to significantly reduce the starch content in the roots of two rice varieties. However, the application of zinc was able to increase the root starch content in both varieties. This effect can be attributed to the higher rate of starch synthesis in rice roots compared to starch decomposition, thereby facilitating starch accumulation. Additionally, the utilization of zinc aids in the transportation of assimilated products from the source leaf to the underground section, thereby promoting the conversion of sucrose into starch within the root system. When plants are exposed to saline-sodic stress, an increase in root starch content can be advantageous. This increase not only helps plants resist stress but also provides more energy storage for them during times of stress [19]. It helps to maintain normal growth ability. Furthermore, the addition of zinc helps to promote the transport of photosynthates from source leaves to roots (reservoir) in rice, particularly under saline-sodic stress. This leads to an increase in the starch content found in rice roots.

In summary, saline-sodic stress significantly impacts the transport and distribution of photosynthetic products in rice leaves, leading to their accumulation in the source leaves and consequent feedback inhibition of photosynthesis. The addition of zinc, however, mitigates the accumulation of soluble sugar and starch in leaves, facilitating the transport of assimilation products to underground parts. This not only enhances rice photosynthesis but also boosts the plant's resilience saline-sodic stress.

**Fig. 10:**
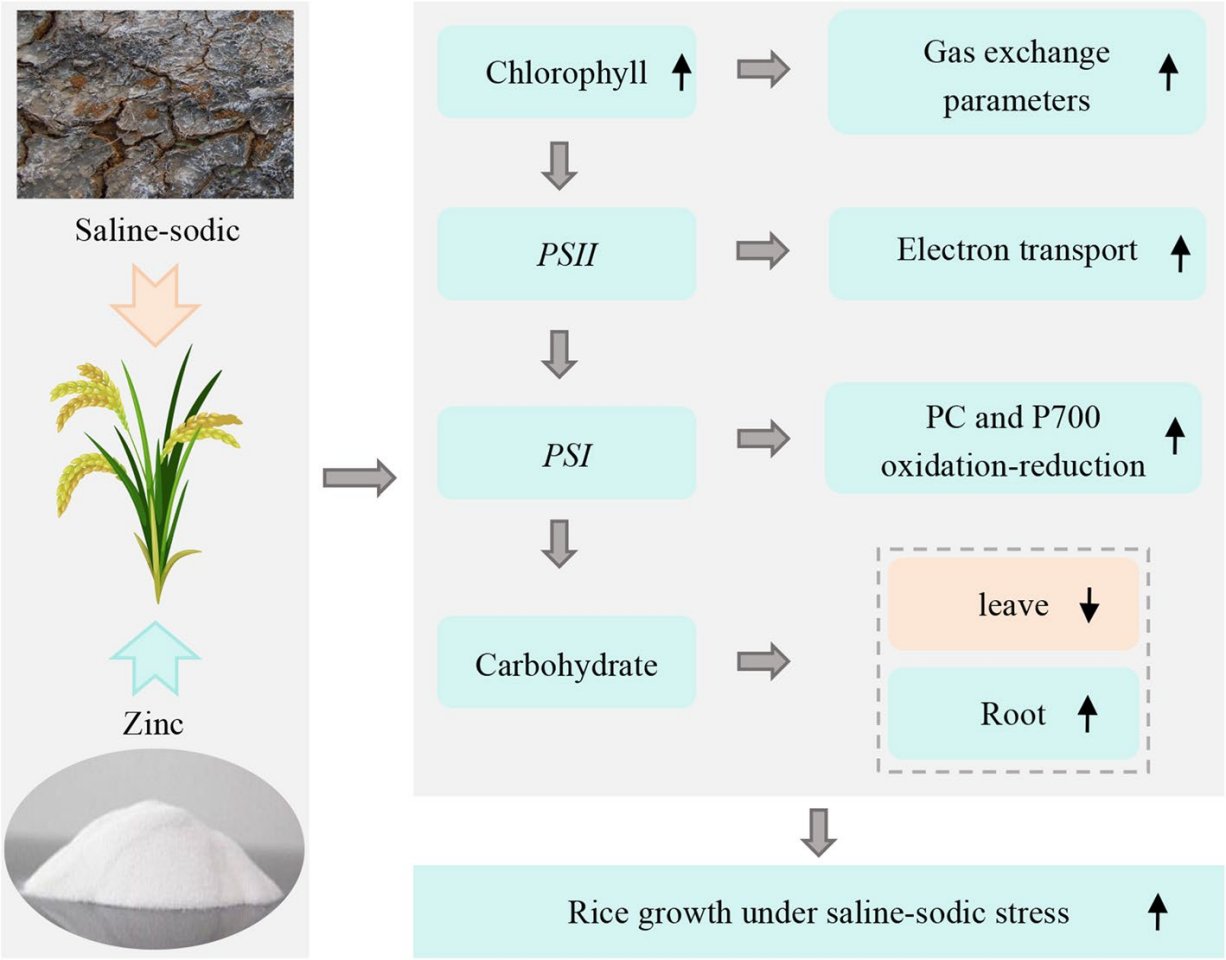
Zinc regulation of rice seedling growth under saline-sodic stress. Black upward and downward arrows indicate the increase and decrease in each indicator

In short, zinc deficiency is a well-known issue in saline-sodic rice fields, but the application of exogenous zinc effectively addresses this issue. Figure 10 illustrates that introducing exogenous zinc in saline-sodic rice areas not only resolves the lack of available zinc but also mitigates the damage inflicted by saline-sodic stress on rice plants. This intervention not only aids in pigment synthesis in rice leaves but also enhances electron transfer in the photosynthetic system, improves carbohydrate metabolism, ultimately influencing the growth and development of rice plants in saline-sodic rice fields.

## Conclusion

Photosynthesis is a fundamental process in plant growth and development. Saline-sodic stress can have a detrimental impact on photosynthesis in rice, leading to decreased yield. This study found that zinc had a positive influence on the growth of two rice varieties under saline-sodic stress, with 'Tonghe 899' showing a more pronounced response. When exposed to saline-sodic stress, the application of exogenous zinc enhances the pigment content in rice leaves, improves the performance of the *PSII* donor/acceptor side (*W*_K_ and *V*_J_), and increases the redox rate of *PSI* (*V*_ox_ and *V*_red_). It also repairs the photosynthetic electron transport chain and promotes the balance of energy distribution. Moreover, zinc application not only decreases the accumulation of soluble sugar and starch in rice leaves in saline-sodic regions, reducing the feedback inhibition of photosynthesis, but also facilitates the transport of assimilation products to the underground parts of the plant. This provides more energy for root growth under saline-sodic stress, enhancing the saline-sodic resistance of rice and promoting the overall growth and development of the plants.

## Data Availability

The data that support the findings of this study are available on request from the corresponding author.

## Abbreviations

*ROS*: Reactive oxygen species
*RWC*: Relative water content
*Pn*: Net photosynthetic rate
*Gs*: Stomatal conductance
*PQ*: Plastoquinone
*OEC*: Oxygen extraction complex
*Fdx*: Ferredoxin
*FNR*: Ferredoxin NADP^+^ reductase
